# Impact of Solvent Properties of Cold-Pressed and Steam-Distilled Orange Oils on GuttaFlow2 and Gutta-Percha

**DOI:** 10.7759/cureus.68261

**Published:** 2024-08-31

**Authors:** Idil Ozden, Mustafa Enes Özden, Hesna Sazak Ovecoglu

**Affiliations:** 1 Endodontics, Marmara University Faculty of Dentistry, Istanbul, TUR; 2 Public Health, Republic of Türkiye Ministry of Health, Kahramankazan District Health Administration, Ankara, TUR

**Keywords:** gutta-percha solvents, guttaflow2, endodontics, root canal retreatment, orange oil

## Abstract

Introduction: Root canal retreatment often employs organic solvents like chloroform, eucalyptol, and orange oil. However, studies comparing their effectiveness yield inconsistent results. The quantity of d-limonene, a crucial component in orange oil, varies depending on the oil production method. Cold-pressed orange oil has been observed to contain the highest d-limonene levels. This study investigates the comparative solvent effects of cold-pressed and steam-hydrodistilled orange oils on gutta-percha and GuttaFlow2, typically used components in root canal fillings.

Methods: Thirty-two discs (10 mm in diameter and 2 mm in thickness) were prepared using GuttaFlow and gutta-percha cones. The samples were weighed and then randomly divided into four groups (n=8) based on the type of solvent used. Each group was immersed in its respective solvent for 10 minutes. After exposure to the solvent, the samples were reweighed to determine the amount of material removed.

Results: The weight loss in the group treated with cold-pressed orange oil on gutta-percha was significantly higher than in other groups (GuttaFlow2 + cold-pressed orange oil, gutta-percha + steam hydrodistilled orange oil, GuttaFlow2 + steam hydrodistilled orange oil) (p<0.001, p<0.001, and p<0.001).

Conclusion: According to the study findings, cold-pressed orange oil demonstrated a higher solvent effect on both GuttaFlow2 and traditional gutta-percha compared to steam-hydrodistilled orange oil. This indicates the significant impact of the production method of orange oil on its efficacy as a solvent in root canal therapy retreatment.

## Introduction

Guttapercha, with a long history in endodontic treatment, remains the most commonly used material for root canal filling [1]. It meets basic requirements in endodontic treatment, such as easy removal when needed. However, researchers continue to explore alternative root canal filling materials due to reasons like pressure sensitivity, insufficient hardness, and lack of adhesive properties [2]. GuttaFlow2, introduced as an alternative to gutta-percha, is a silicone-based root canal filling material containing gutta-percha particles and polydimethylsiloxane. The manufacturers claim that this material’s excellent sealing ability is due to its increased flowability and slight expansion during treatment, along with its easy removability from the canal when required [3]. Studies indicate that GuttaFlow2 leaves less residue in the canal during mechanical retreatment procedures as compared to traditional gutta-percha [4].

In cases of failed root canal treatment, retreatment becomes the primary treatment option, which entails numerous mechanical, thermal, and chemical methods and specialized instruments [5,6]. It has been reported that the retreatment procedure takes longer than the initial treatment [7]. The use of various chemical solvents in root canal retreatment can expedite the process. The most commonly used organic solvents include chloroform, xylene, halothane, eucalyptol, turpentine, and orange oil [8]. Despite many studies comparing the effectiveness of these chemicals [8-11], there are few studies in the literature that compare the use of a specific solvent and evaluate its effect on gutta-percha and GuttaFlow [12].

Orange oil, one of the most widely used essential oils in the world, can be produced by different methods such as cold pressing and steam hydrodistillation [13]. Research has shown that the main component of orange oil is d-limonene and that production methods can lead to variations in the oil composition [3,14].

This study hypothesizes that different orange oil production techniques may have different solvent effects on traditional gutta-percha and GuttaFlow2 when used as solvents. This study aimed to compare the solvent effects of orange oils obtained by two different production methods on gutta-percha and GuttaFlow2 discs.

## Materials and methods

Sample preparation

Thirty-two discs (10 mm in diameter and 2 mm in thickness) were prepared using GuttaFlow and gutta-percha cones. The samples were weighed and then randomly divided into four groups (n=8) based on the type of solvent used. In determining the number of disks used in the research, the quota sampling method was used, considering the budget and the sample sizes of other research in the literature [9,10].

**Figure 1:**
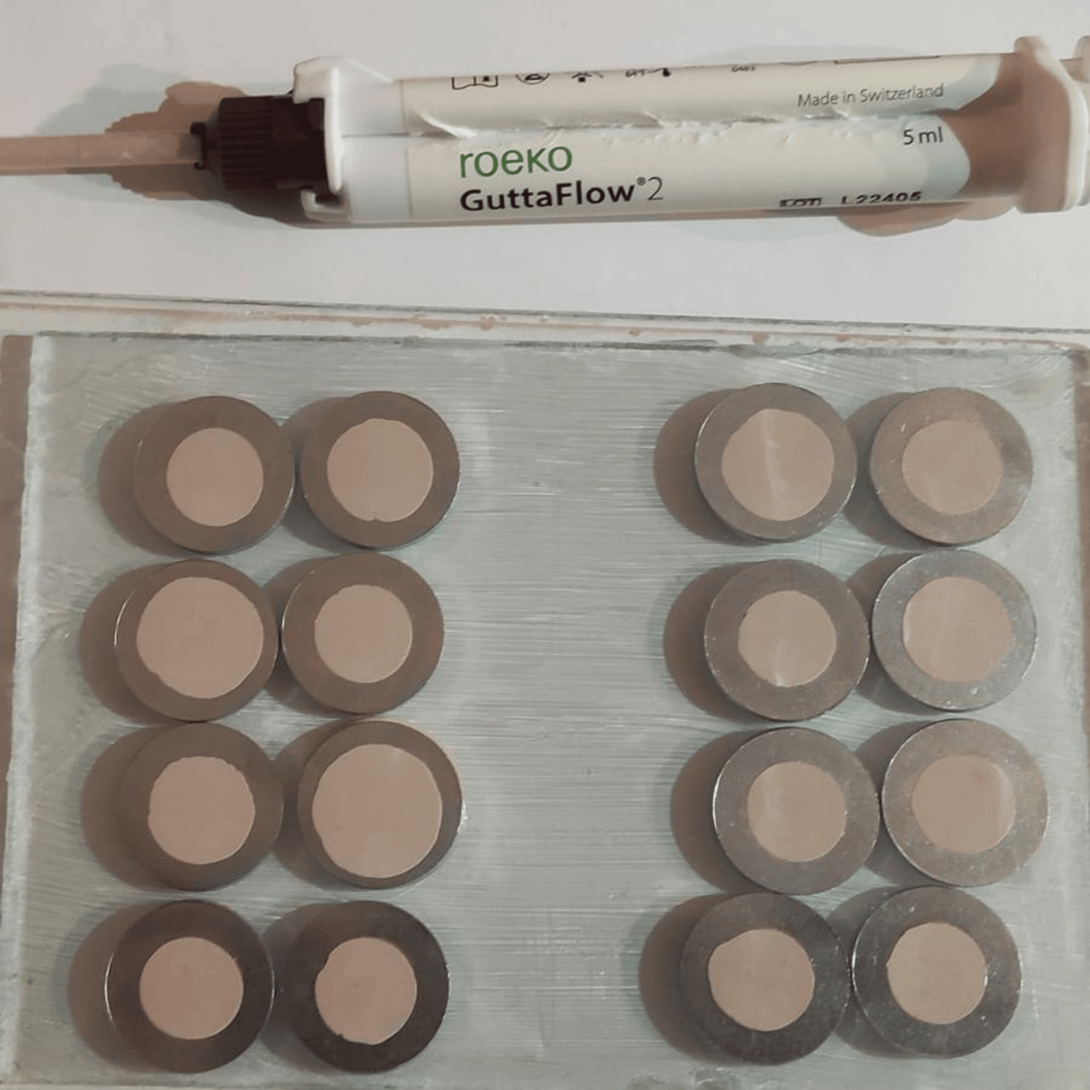
GuttaFlow2 samples (experimental groups 1 and 2).

**Figure 2:**
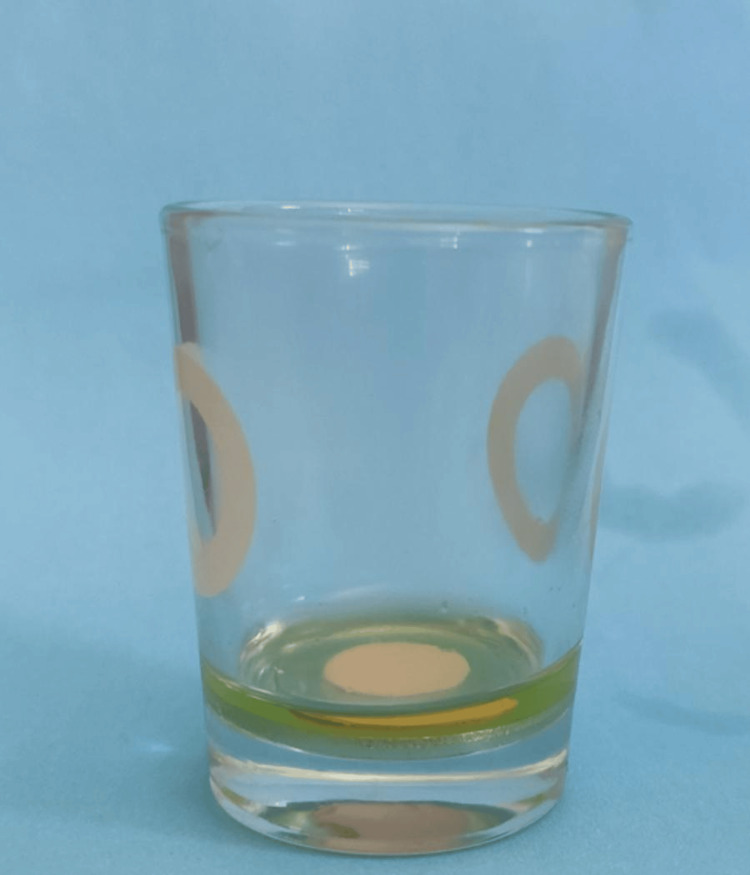
Contact with solvents (solvents application).

In this study, pure orange oil produced by the cold pressing method (Antalya, Turkey: Gençay Ltd. Co.) and pure orange oil produced by the steam hydrodistillation method (Istanbul, Turkey: Arifoğlu Spices and Food Industry. Trade Ltd.) were utilized as solvents. GuttaFlow2 (Langenau, Germany: Coltene/Whaledent, Inc.) and traditional gutta-percha cones (Ho Chi Minh City, Vietnam: Pearl Dent Co., Ltd.) were employed as root canal filling materials. Metal rings with a diameter of 10 mm and a thickness of 2 mm were used in the experimental groups to ensure equal volumes of root canal filling materials and equal surface areas in contact with the solvents (Figures 1, 2).

A total of 16 discs were prepared for each type of root canal filling material by placing them inside the metal rings. To prepare the gutta-percha test specimens, traditional gutta-percha cones were softened by immersion in water at 70°C for 1 minute. The softened gutta-percha cones were then placed inside the metal rings and compressed between two glass plates for 1 minute. For the preparation of other group specimens, GuttaFlow2 was applied inside the metal rings according to the manufacturer's instructions. The metal rings were compressed between two glass plates for 30 minutes. All samples were stored at 37°C for 24 hours. After 24 hours, each sample was weighed using an electronic precision balance (Precisa XB 220 A, Precisa Gravimetrics AG, Dietikon, Switzerland). Each weight measurement was repeated 5 times, and the average of the measurements was calculated for each sample. The average value obtained was recorded as the initial weight.

Experimental groups

The samples were randomly divided into the following four groups based on the type of orange oil used (n=8): group 1 - GuttaFlow2 + cold pressed orange oil, group 2 - GuttaFlow2 + steam hydrodistilled orange oil, group 3 - Traditional gutta-percha + cold pressed orange oil, and group 4 - traditional gutta-percha + steam hydrodistilled orange oil.

Each GuttaFlow2 and traditional gutta-percha sample in the experimental groups was immersed for 10 min in glass containers containing 2 mL of solvent. The samples were then kept in distilled water for 20 min to neutralize the effect of the solvent. After removal from the distilled water, the samples were kept at 37°C for five days to allow the remaining distilled water and solvent to evaporate. The final weight at the end of the fifth day was recorded as the final weight. The differences between the initial and final weights of the discs were recorded as the amount dissolved and removed by the solvents.

Statistical analyses

Statistical analyses were performed using SPSS version 29 (Armonk, NY: SPSS Corp.). Descriptive variables were expressed as median and interquartile range. The weight loss caused by orange oils was compared between groups using the Kruskal-Wallis test. The overall type 1 error level was used for statistical significance at 5%, and the significance level was set at p<0.001.

## Results

**Figure 3:**
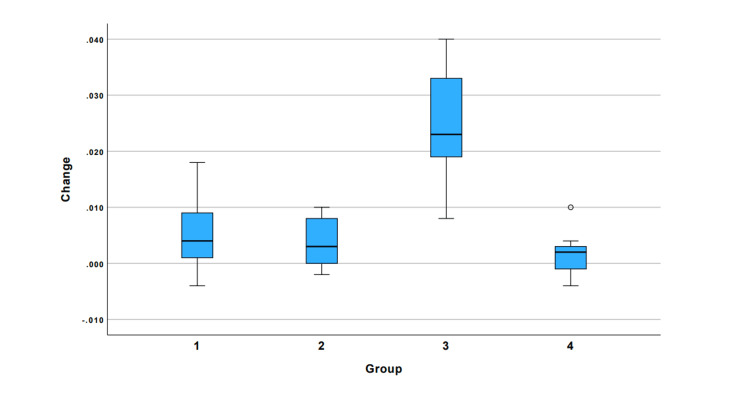
Distribution of change based on experimental groups. Group 1: GuttaFlow2 + cold pressed orange oil, group 2: GuttaFlow2 + steam hydrodistilled orange oil, group 3: traditional gutta-percha + cold pressed orange oil, and group 4: traditional gutta-percha + steam hydrodistilled orange oil

**Figure 4:**
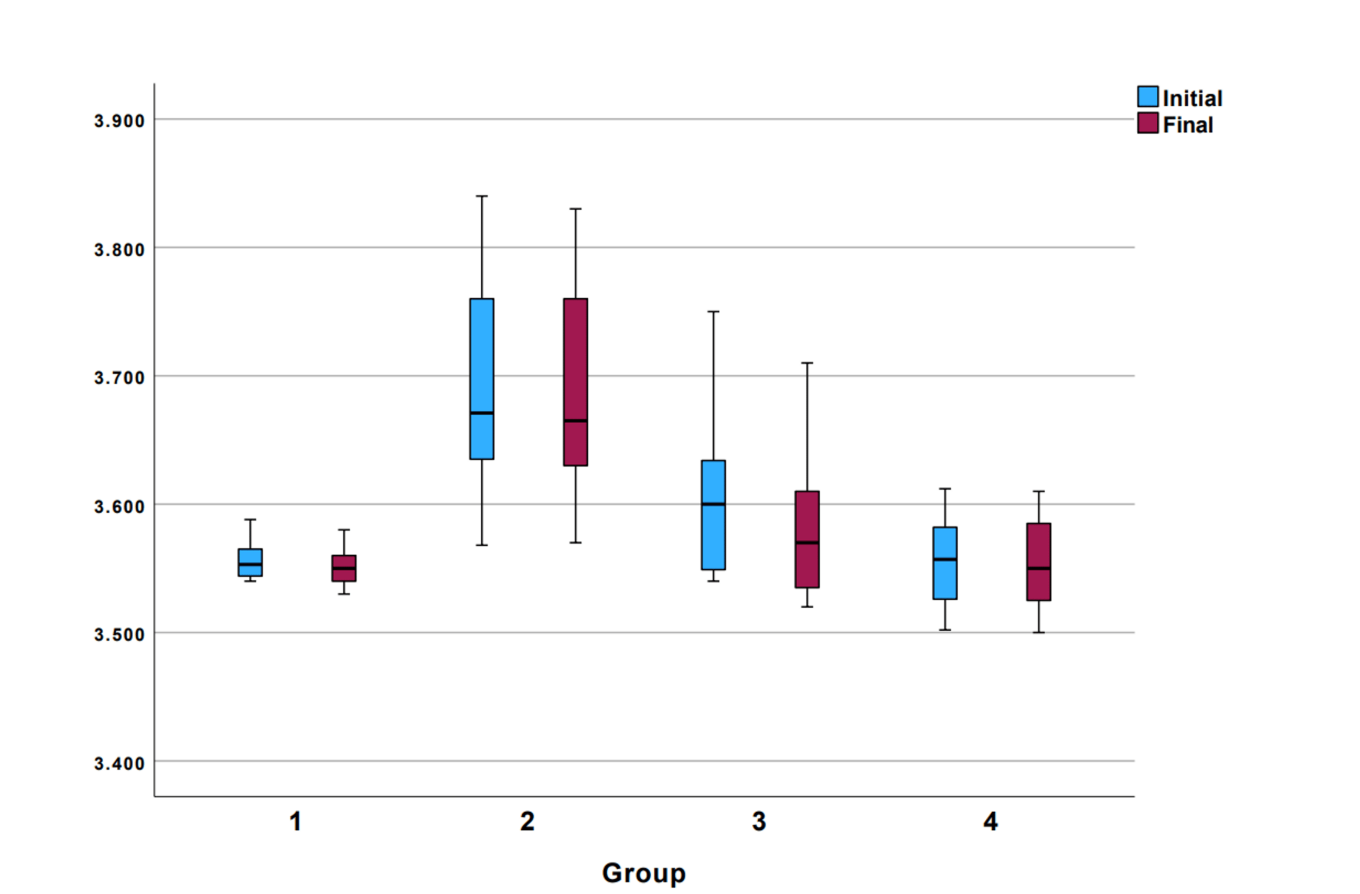
Distribution of starting and final measurements based on experimental groups. Group 1: GuttaFlow2 + cold pressed orange oil, group 2: GuttaFlow2 + steam hydrodistilled orange oil, Group 3: traditional gutta-percha + cold pressed orange oil, and group 4: traditional gutta-percha + steam hydrodistilled orange oil

The distribution of initial and final measurements across the groups is presented in Figure 3. The average amount of material removed by the solvents over the specified time interval is shown in Figure 4. The pairwise comparisons showed that the weight loss achieved in group 3 was statistically significantly higher than the other three groups (p<0.001) (Table 1).

**Table 1:** Comparisons of initial and final measurements. *P-values calculated using the Kruskal-Wallis test. IQR: interquartile range; Min-max: minimum-maximum values.

Group	Median (IQR)	Min-max	p-Value*
Initial measurement (mg)			
G1 (n=8)	3.553 (0.024)	3.540-3.588	0.003
G2 (n=8)	3.671 (0.144)	3.568-3.840	
G3 (n=8)	3.600 (0.091)	3.222-3.750	
G4 (n=8)	3.557 (0.065)	3.502-3.612	
Final measurement (mg)			
G1 (n=8)	3.550 (0.020)	3.530-3.580	0.003
G2 (n=8)	3.665 (0.150)	3.570-3.830	
G3 (n=8)	3.570 (0.088)	3.200-3.710	
G4 (n=8)	3.550 (0.070)	3.500-3.610	
Difference (mg)			
G1 (n=8)	0.004 (0.009)	-0.004-0.018	0.001
G2 (n=8)	0.003 (0.008)	-0.002-0.010	
G3 (n=8)	0.023 (0.016)	0.008-0.040	
G4 (n=8)	0.002 (0.005)	-0.004-0.010	

When the dissolution values of traditional gutta-percha and GuttaFlow2 in the solvents were compared, no statistically significant result was obtained. Although not statistically significant, the solvent effect of orange oil obtained by cold pressing was found to be higher in both the traditional gutta-percha and GuttaFlow groups than that of orange oil obtained by steam hydrodistillation.

## Discussion

Gutta-percha, a trans-isomer of polyisoprene, is a solid root canal-filling material. It was introduced to the endodontic community by Bowman in 1867. Since its introduction, it has served as the most widely used root canal filling material due to its compressibility, non-staining of dentin, and the possibility of root canal retreatment.

GuttaFlow2, introduced as a cold flowable filling system combining gutta with polyisoprene, contains gutta-percha particles that are 30 μm smaller and can expand by 0.2% during treatment. It is therefore considered to be the almost impermeable agent that can create a tighter seal [15].

The failure of root canal treatment necessitates endodontic retreatment. During the endodontic retreatment, the old root canal filling and paste should be removed from the root canal system as much as possible for effective cleaning of any remaining tissue and bacteria in the root canal system [16].

Much effort has been put into finding a practical method of removing gutta-percha from the canal. Methods used to remove gutta-percha from the canal can be thermal (using heat-conducting systems), mechanical (using hand instruments), chemical (using various solvents), or a combination of these three techniques [17,18]. Chemical retreatment involves the use of various solvents, such as xylene, chloroform, methylene chloride, eucalyptol oil, and orange oil [19].

Orange oil is an essential oil with a pleasant fragrance that acts as an antispasmodic, sedative, antiinflammatory, and antiseptic. Due to its safety, biological compatibility, and lack of harmful effects, its ability to dissolve gutta-percha has been investigated since the early 1990s [20]. Despite many studies suggesting that orange oil has a solvent effect similar to chloroform and eucalyptol as alternatives to gutta-percha solvents, some studies also indicate that its effect may be weaker [21]. Similarly, while some reports present orange oil as a superior solvent to eucalyptol, others offer inconsistent results showing no difference [22].

The literature review demonstrates that the retreatment procedures in studies using GuttaFlow are often performed with mechanical systems [4,23]. However, there is only one study in the literature that evaluates the effect of gutta-percha solvents on GuttaFlow [12].

The objective of this study was to compare the solvent effects of orange oils, obtained through two distinct production methods - on GuttaFlow and traditional gutta-percha. We adopted the method described by Whitworth et al. as the reference for evaluating the solvent effect [24]. This involved calculating the amount of material mixed with distilled water post-dissolution. Despite the absence of a universally accepted gold standard for assessing solvent effects, the current study employed this method due to its practicality, repeatability, and cost-effectiveness. However, the limitations of this method include its inability to fully simulate clinical conditions and its failure to account for material softening [25].

Orange oil is an essential oil that has been demonstrated to exert a multitude of salutary effects on human health. It has been demonstrated that this essential oil possesses antioxidant, antiinflammatory, and antimicrobial properties, which collectively support the immune system, protect skin health, and alleviate stress [26]. Furthermore, it has a soothing effect on the digestive system and plays a supportive role in the treatment of respiratory infections [27]. Due to these properties, orange oil is widely used in aromatherapy, cosmetics, and the food industry. In addition to its general health benefits, orange oil is also utilized in endodontics as a solvent, particularly in the removal of root canal filling materials, due to its high limonene content. The most important characteristic of orange oil rendering it an effective solvent is its high limonene content [28]. The d-limonene content of orange oil can vary depending on factors such as the species of plant from which it is obtained, the drying process employed after harvesting, and the oil extraction technique [29,30]. The primary methods employed for the extraction of orange oil are cold pressing and distillation. The process of cold pressing entails the mechanical pressing of orange peels with the objective of releasing the oil. This method preserves the oil's natural aroma and nutritional properties, as it does not involve the application of heat. In contrast, the distillation process, which is typically steam distillation, involves heating the orange peels with steam, which causes the volatile oils to evaporate. Subsequently, the steam is condensed, resulting in the collection of the oil. Nevertheless, the application of distillation may occasionally result in the production of an oil of inferior quality, due to the potential degradation of certain fragile compounds caused by the heat employed. Gölükcü et al.’s study demonstrates that orange oil obtained by the cold pressing method contained significantly higher d-limonene content than orange oil obtained by steam hydrodistillation [15].

In accordance with the prior obtained results, the null hypothesis of this study was accepted. The evaluated solvent effect was higher in the group where orange oil obtained by cold pressing was applied to traditional gutta-percha discs and lower in the group where orange oil obtained by steam hydrodistillation was applied to GuttaFlow discs. The group using cold-pressed orange oil on traditional gutta-percha exhibited significantly higher weight loss than the other three groups (cold-pressed orange oil-GuttaFlow; steam hydrodistillation-traditional gutta-percha; steam hydrodistillation-GuttaFlow).

Previous studies investigating the effect of gutta-percha solvents on traditional gutta-percha have found that orange oil has a similar solvent effect to chloroform and eucalyptol, but there are also results indicating a lesser effect. In the absence of information regarding the production methods of the orange oils used in these studies, it is challenging to draw any firm conclusions. However, the inconsistent results may be attributed to the different production methods of the orange oils used.

Limitations of this study include the inability of the samples used to fully simulate clinical conditions in terms of size and contact area and the inability of the evaluation method used in the study to account for softening. Furthermore, the potential variations introduced by trace elements in the orange oils in question were not considered, and a comparison with pure d-limonene was not conducted. In light of these limitations, this study can be regarded as a valuable point of reference for future research on the content and production methods of orange oil commonly used in clinical practice. Further clinical studies in this field are strongly recommended.

## Conclusions

Beyond the limitations of this study, it was observed that orange oil obtained by cold pressing had a higher solvent effect on both GuttaFlow2 and traditional gutta-percha than orange oil obtained by steam hydrodistillation. Therefore, the use of orange oil in non-surgical root canal retreatment is suitable for both types of restorative materials. However, further in vivo and in vitro studies are needed to evaluate this issue in more detail, along with detailed information on the production methods of the orange oils used in the studies. Addressing these limitations and conducting further research can yield a more comprehensive interpretation of orange oil's potential as a solvent in root canal retreatment.
